# HMGB1 and Toll-like receptors: potential therapeutic targets in autoimmune diseases

**DOI:** 10.1186/s10020-023-00717-3

**Published:** 2023-09-04

**Authors:** Wenxuan Ren, Lei Zhao, Ying Sun, Xichang Wang, Xiaoguang Shi

**Affiliations:** 1 Department of Endocrinology, Shengjing Hospital of China Medical University, Shenyang, 110001 Liaoning China; 2 Department of Laboratory Medicine, The First Hospital of China Medical University, Shenyang, 110001 Liaoning China

**Keywords:** HMGB1, Toll-like receptors, Autoimmune diseases, Rheumatoid arthritis, Systemic lupus erythematosus, Autoimmune thyroid disease

## Abstract

HMGB1, a nucleoprotein, is expressed in almost all eukaryotic cells. During cell activation and cell death, HMGB1 can function as an alarm protein (alarmin) or damage-associated molecular pattern (DAMP) and mediate early inflammatory and immune response when it is translocated to the extracellular space. The binding of extracellular HMGB1 to Toll-like receptors (TLRs), such as TLR2 and TLR4 transforms HMGB1 into a pro-inflammatory cytokine, contributing to the occurrence and development of autoimmune diseases. TLRs, which are members of a family of pattern recognition receptors, can bind to endogenous DAMPs and activate the innate immune response. Additionally, TLRs are key signaling molecules mediating the immune response and play a critical role in the host defense against pathogens and the maintenance of immune balance. HMGB1 and TLRs are reported to be upregulated in several autoimmune diseases, such as rheumatoid arthritis, systemic lupus erythematosus, type 1 diabetes mellitus, and autoimmune thyroid disease. The expression levels of HMGB1 and some TLRs are upregulated in tissues of patients with autoimmune diseases and animal models of autoimmune diseases. The suppression of HMGB1 and TLRs inhibits the progression of inflammation in animal models. Thus, HMGB1 and TLRs are indispensable biomarkers and important therapeutic targets for autoimmune diseases. This review provides comprehensive strategies for treating or preventing autoimmune diseases discovered in recent years.

## Introduction

High-mobility group (HMG) proteins, which are non-histone DNA-binding proteins, are expressed in eukaryotic cells and exhibit various biological functions (Dumitriu et al. 2005). HMGB1, which is the most abundant member of the HMG family (Ge et al. 2021), is abundantly expressed in almost all human cells. Additionally, HMGB1 can actively and passively shuttle between the nucleus and the cytoplasm of all cells (Kang et al. 2014). HMGB1 is mainly localized to the nucleus where it interacts with DNA and regulates chromosome structure, maintains the integrity of nucleosomes, and promotes gene transcription. During biological processes, such as apoptosis, necrosis, and cell scorching, HMGB1 is transferred to the extracellular space (Chen et al. 2022). In the extracellular environment, HMGB1 can function as a damage-associated molecular pattern (DAMP) or alarm protein (alarmin) and mediate early inflammatory and immune response (Kang et al. 2014). Previous studies have reported that HMGB1 functions as an important inflammatory cytokine in various autoimmune diseases. Especially in rheumatoid arthritis (RA) (Schierbeck et al. 2013), systemic lupus erythematosus (SLE) (Wirestam et al. 2015), and autoimmune thyroid disease (AITD) (Li et al. 2017). Toll-like receptors (TLRs) are members of a family of pattern recognition receptors and can express on various cells, such as monocytes, macrophages, dendritic cells, and B cells (Iwasaki and Medzhitov 2004). Furthermore, TLRS can function as pathogen-associated molecular models (PAMPs), bind to endogenous DAMPs and activate the innate immune response (Chuang and Ulevitch 2001). Some pathogenic microorganisms, such as bacteria, viruses and fungi, can activate TLR signaling pathway, trigger innate immune response and promote the release of inflammatory mediators (Ulevitch 2004). Generally, TLRs are key signaling molecules mediating the immune response and play a critical role in the host defense against pathogens and the maintenance of immune balance. Previous studies have demonstrated that TLRs are also involved in the occurrence and development of various autoimmune diseases, such as RA (Drexler and Foxwell 2010), SLE (Wen et al. 2023), type 1 diabetes mellitus (T1DM) (Dandona et al. 2013), and AITD (Peng et al. 2016). HMGB1 can bind to TLRs and other PRRs to activate the TLR signaling pathway and induce cytokine production and immune cell proliferation, promoting the production of pro-inflammatory cytokines and eliciting inflammatory responses. The potential of HMGB1 and TLRs as therapeutic targets has been previously examined.

This review summarized the structural, functional, and immunological characteristics of HMGB1 and TLRs and the key roles of HMGB1 and TLRs in development of autoimmune diseases. Additionally, recent advances in establishing HMGB1 and TLRs as therapeutic targets for autoimmune diseases have been discussed.

## HMGB1: origin and structure

In 1973, the research group of Ernest Johns and Graham Goodwin successfully extracted an abundant non-histone nuclear protein from calf thymus chromatin. This protein was named "high-mobility group" protein (Goodwin et al. 1973) owing to its rapid migration in polyacrylamide gel electrophoresis without aggregation (Goodwin and Johns 1973). This was the first time that HMGB1 was discovered. In 2001, Michael Bustin reclassified these proteins into three superfamilies and renamed them HMGA, HMGB and HMGN (Bustin 2001). Among them, the high-mobility group box (HMGB) family is the most abundant protein family of HMGs (Goodwin and Johns 1978). HMGB is highly conserved and comprises four members (HMGB1, HMGB2, HMGB3, and HMGB4). In 2001 and 2003, the homologs of mammalian HMGB1 were identified in bacteria, drosophila, echinoderms, plants, and fish (Bustin 2001; Wu et al. 2003). HMGB1 mRNA is polyadenylated (Bustin et al. 1981). Meanwhile, the homology of HMGB1 protein sequence between mice and rats is 100%, while that between rodents and humans is 99% (Ferrari et al. 1994; Wen et al. 1989).

**Fig. 1:**
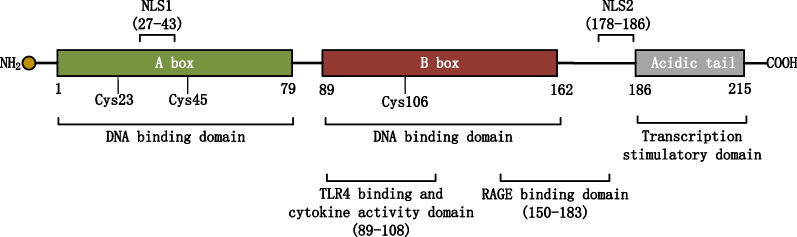
The structure and function of HMGB1. HMGB1 contains two DNA binding domains (HMG A box domain [9–79 aa] and HMG B box domain [95–163 aa]), a C-terminal acidic tail (186–215 aa) and a N-terminal region. Residues 150–183 bind to RAGE to promote cell migration, while residues 89–108 in charge of activating cytokine and binding to the TLR4 structural domain to promote inflammation. Two nuclear localization signals: NLS1 (28–44 aa) and NLS2 (179–185 aa). HMGB1 encode three cysteines: Cys23, Cys45 and Cys106

Human HMGB1, which comprises 215 amino acid residues, contains two DNA-binding domains (HMG A box domain [9–79 amino acids] and HMG B box domain [95–163 amino acids]), a C-terminal acidic tail (186–215 amino acids) (Bianchi et al. 1992) (Fig. 1), and a functionally important N-terminal region. The DNA-binding domain contains nuclear export signals (NESs) (Tang et al. 2014). The homeostasis of HMGB1 in the nucleus is dependent on two nuclear localization signals (NLSs) (NLS1 [28–44 amino acids] and NLS2 [179–185 amino acids]) (Bonaldi et al. 2003). The localization of HMGB1 is disrupted upon alterations in NES and NLS. HMGB1 can bind to various proteins. This binding is critical for the activity and function of HMGB1. For example, the residues 150–183 bind to RAGE to promote cell migration (Huttunen et al. 2002), while the residues 89–108 bind to the TLR4 structural domain to promote inflammation (Venereau et al. 2016). The extracellular B box exhibits pro-inflammatory activity, whereas the A box functions as an antagonist of HMGB1. The A box can bind to the B box and exert anti-inflammatory effects (Li et al. 2003). The structures of two HMG boxes of HMGB1 are similar to those of a typical DNA-binding domain, comprising three alpha helices arranged in an "L" shape and two loops (Hardman et al. 1995; Thomas and Travers 2001). Three lysine residues are encoded at positions 23, 45, and 106 of HMGB1 (Hoppe et al. 2006). Generally, the structure of HMGB1 is highly conserved among species.

## Mechanism of HMGB1 release

In 1999, after treating mouse macrophages with endotoxin lipopolysaccharide (LPS), Wang et al. found that large amounts of HMGB1 were secreted into the extracellular space (Wang et al. 1999). This was the first study to report that HMGB1 is a secretory protein (Xue et al. 2021). Additionally, this study highlighted the role of extracellular HMGB1 in inflammation and infection. In 2003, the research group of Tiziana Bonaldi and Marco Bianchi, for the first time, demonstrated that the acetylation and deacetylation of NLS1 and NLS2 promote the rapid shuttling of HMGB1 from the nucleus to the cytoplasm and consequently promote the secretion of HMGB1 from activated monocytes (Bonaldi et al. 2003). Inflammatory stimuli promote the acetylation of lysine residues in HMGB1, preventing the nuclear entry of HMGB1 (Bonaldi et al. 2003), which is critical for its translocation from the nucleus to the cytoplasm (Gardella et al. 2002; Rendon-Mitchell et al. 2003). Additionally, the phosphorylation of serine residues in NLS1 and NLS2 promotes the translocation of HMGB1 from the nucleus to the cytoplasm (Bonaldi et al. 2003; Wang et al. 2019). HMGB1 reaches the external cellular environment through active secretion by cells of the innate immune system (Wang et al. 1999; Agnello et al. 2002) or passive release after necrotic cell death (Scaffidi et al. 2002). During apoptosis, necrosis, cell scorching cell death, and injury resulting from various stimuli, such as irradiation, hypoxia, and hyperthermia (Pisetsky 2014), soluble HMGB1 can be released into the extracellular space in large quantities by these dead or dying cells and elicit inflammatory responses in vitro and in vivo (Scaffidi et al. 2002). Several studies have demonstrated that endogenous stimuli, exogenous microbial products, and infections with various pathogens can induce active secretion of HMGB1 by immune cells, endothelial cells, epithelial cells, fibroblasts, or other cells (Magna and Pisetsky 2014; Andersson et al. 2018). The active release of HMGB1 from immune cells also involves two modalities. In the first modality, the activation of target cells by exogenous stimuli leads to the secretion of HMGB1 into the extracellular space (Bonaldi et al. 2003). The second modality involves the lack of a secretory signal peptide for HMGB1, which cannot be transported through the classical endoplasmic reticulum and Golgi apparatus secretion pathways (Gardella et al. 2002; Rendon-Mitchell et al. 2003). This HMGB1 is then packaged into intracellular vesicles (such as lysosomes), which fuse with the cytoplasmic membrane and release HMGB1 into the extracellular space (Pisetsky 2014). Extracellular HMGB1 can function as a DAMP to activate the innate immune system by recruiting inflammatory cells and smooth muscle cells and stimulate macrophages and endothelial cells to produce pro-inflammatory cytokines that promote the inflammatory response (Andersson et al. 2000; Fiuza et al. 2003). Additionally, as an immune molecule, extracellular HMGB1 can trigger the inflammatory response of immune cells and endothelial cells. Subsequently, immune cells and endothelial cells activated by HMGB1 can secrete HMGB1, resulting in the formation of a positive feedback loop (Kang et al. 2014). Thus, HMGB1 can maintain a long-term inflammatory state under a variety of stimulation conditions (Kang et al. 2014).

## TLRs: structure and function

TLRs, which are transmembrane receptors mainly expressed by innate immune cells, are part of a family of pattern recognition receptors (PRRs) utilized by the innate immune system (Luchner et al. 2021). In humans, TLR1, TLR2, TLR4, TLR5, and TLR6 are expressed on the cell surface, while TLR3, TLR7, TLR8, and TLR9 are expressed on the endosomal membrane (Dvornikova et al. 2020). TLRs, an evolutionarily conserved type I transmembrane protein superfamily, comprise a leucine-rich repeat extracellular domain that can interact with PAMPs or DAMPs and a cytoplasmic Toll/interleukin (IL)-1 receptor (TIR) domain that is involved in downstream signal transduction and eliciting inflammatory responses (Botos et al. 2011; Lim and Staudt 2013). Additionally, TLRs are synthesized in the endoplasmic reticulum, processed in the Golgi apparatus, and transported to the plasma membrane based on the localization of PAMPs they recognize (Ullah et al. 2016; Gay et al. 2014). TLRs can recognize various PAMPs, including DAMPs, gram-negative and gram-positive bacteria, viruses, flagellin, and nucleic acids, according to their localization on the cell surface or within the cells (Marongiu et al. 2019). Upon binding to ligands, TLRs undergo homodimerization or heterodimerization and recruit adaptor proteins containing the TIR structural domain to activate the downstream signaling pathways (Luchner et al. 2021). For example, TLRs can activate interferon (IFN)-β (TRIF) signaling through the recruitment of the adapter molecule myeloid differentiation primary response differentiation gene 88 (MyD88) or MyD88-independent TIR domain-containing adapter molecules (Satoh and Akira 2016). This is followed by the activation of nuclear factor kappa-light-chain-enhancer of activated B cells (NF-κB) signaling and mitogen-activated protein kinase (Owen et al. 2020), which promotes the expression of pro-inflammatory cytokines, such as tumor necrosis factor-α (TNF-α) or Interleukin-6 (IL-6) (Kawasaki and Kawai 2014). Additionally, TLRs can activate type I IFN responses mediated by IFN regulatory factors (IRFs), such as IRF3 and IRF7, which play a major role in adaptive immunity (Moynagh 2005; Zhao et al. 2015). Thus, TLRs recognize several danger signals to activate innate immune responses against infection and injury.

## HMGB1 and TLRs

**Fig. 2:**
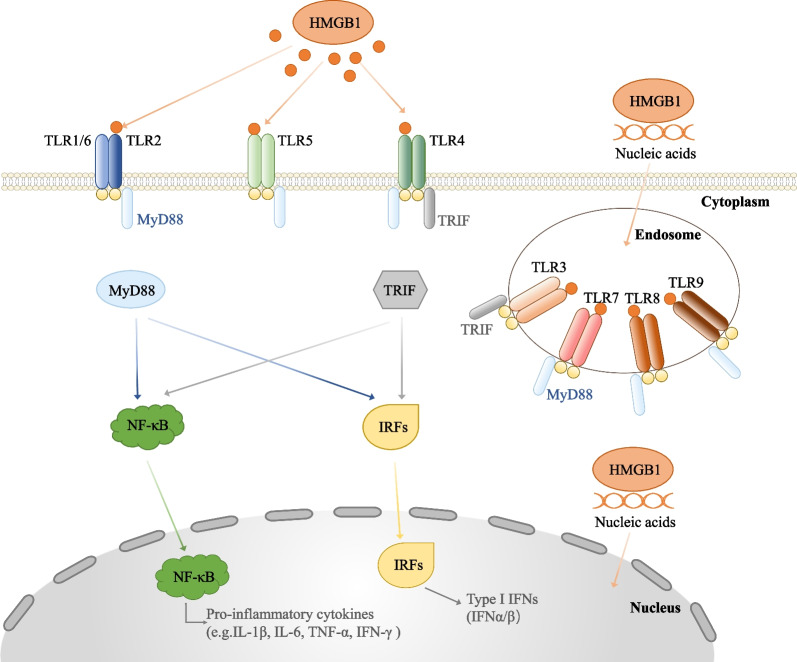
HMGB1 and TLRs signaling pathway. HMGB1 can combined with cell surface TLRs (TLR1, TLR2, TLR4, TLR5, TLR6), while the nucleic acid of HMGB1 can cross the cell membrane and bind to the endosomal TLRs (TLR3, TLR7, TLR8, TLR9). TLRs recruit TIR domain-containing adaptor proteins like MyD88 and TRIF, which activate NF-κB signaling and IRFs. This initiates the expression of pro-inflammatory cytokines such as IL-1β, IL-6, TNF-α, IFN-γ or the type I interferons IFN-α and IFN-β. The figure distinguishes by color the adaptor proteins MyD88 and TRIF that bind to different TLRs. TRIF: TIR domain-containing adaptor protein inducing IFNβ, MyD88: molecule myeloid differentiation primary response differentiation gene 88, NF-κB: nuclear factor kappa-light-chain-enhancer of activated B cells, IRF: Interferon Regulatory Factor, IFN: Interferon, TNF-α: tumor necrosis factor-alpha, IL: Interleukin

HMGB1 can interact with TLRs (TLR2, TLR4, and TLR9) to activate TLR signaling. Upon stimulation by the ligand, TLRs recruit downstream junction molecules, including TIR domain-containing adapter-inducing IFN-β (TRIF) and MyD88, which subsequently activate the NF-κB and IRF signaling pathways, promote the production of cytokines and chemokines involved in immune responses, and induce inflammation (Beutler 2004). TLR4 signaling is essential for HMGB1-induced cytokine production. Macrophages lacking TLR4 cannot release pro-inflammatory cytokines in response to HMGB1 (Li et al. 2020; Zhang et al. 2021). The intramolecular amino acids C23, C45, and C106 of HMGB1 are required for TLR4 binding and activation (Yang et al. 2012). The TLR4 structural domain can bind to the residues 89–108 of the HMGB1 B box to promote inflammation. The knockdown of *TLR4* in vitro or in vivo mitigates HMGB1-induced tissue damage (Laird et al. 2014), inflammation (Zong et al. 2013), and immune response. In addition to TLR4, HMGB1 also requires TLR2 to promote tissue damage and inflammatory responses (Herzog et al. 2014). However, TLR9 mediates immune responses against nucleotides induced by the HMGB1-DNA complex. As HMGB1 interacts with multiple TLRs, it is a key protein for innate immunity. The specific pathways of HMGB1-induced TLR signaling are described below (Fig. 2).

## Autoimmune diseases

### Rheumatoid arthritis

Rheumatoid Arthritis (RA) is an autoimmune disease that is closely related to HMGB1. HMGB1 is reported to be an important factor in the development of RA (Li et al. 2011; Oktayoglu et al. 2013). RA is characterized by chronic synovial inflammation, articular cartilage erosion and damage, synovial cell proliferation, and bone destruction (Chen et al. 2013). Several studies examining the correlation between RA and HMGB1 or TLRs have demonstrated that HMGB1 and TLRs play an indispensable role in the progression of RA as pro-inflammatory cytokines (Kokkola et al. 2002; Taniguchi et al. 2003; Elshabrawy et al. 2017).

In 2002, the research group of Kokkola first reported that HMGB1 was upregulated in the nucleus, cytoplasm, and extracellular environment of the synovial tissue (ST) and synovial cells in patients with RA and experimental arthritis rat models (Kokkola et al. 2002). In 2003, Taniguchi et al. reported that the serum and ST levels of HMGB1 in patients with RA are higher than those in healthy human controls. Stimulation with HMGB1 promotes the release of pro-inflammatory cytokines, such as TNF-α, IL-1β, and IL-6 from synovial fluid macrophages. Meanwhile, TNF-α stimulation upregulated HMGB1 expression and shifted the localization of HMGB1 from the nucleus to the cytoplasm, which further confirmed the key role of HMGB1 in the pathogenesis of RA and suggested the presence of a pro-inflammatory circuit between HMGB1 and TNF-α (Taniguchi et al. 2003). Pullerits et al. demonstrated that the injection of recombinant HMGB1 protein into mice induced arthritis (Pullerits et al. 2003). Other studies have demonstrated that the suppression of HMGB1 expression or treatment with therapeutic agents (including polyclonal and monoclonal anti-HMGB1 antibodies, the A-frame structural domain of recombinant HMGB1, soluble RAGE, and corticosteroids) can inhibit the progression of RA (Palmblad et al. 2007; Af Klint et al. 2005; Hofmann et al. 2002; Wouwer et al. 2006; Zetterström et al. 2008; Hamada et al. 2008). Intra-articular corticosteroid injection has been shown to suppress the extracellular effect of HMGB1 in RA patients (Af Klint et al. 2005). In experimental RA animal models, the suppression of HMGB1 expression alleviates cartilage and bone damage (Ostberg et al. 2010; Schierbeck et al. 2011). Additionally, HMGB1 can promote the activation and polarization of Th17 cells by upregulating TLR2 and Th17 cell-related cytokines in patients with RA. This suggests that the increase of Th17 cells may partly contribute to HMGB1-mediated immune dysregulation in RA (Shi et al. 2012; He et al. 2012). Samarpita et al. (2020) reported that in the adjuvant-induced arthritis rat model, TAK-242(an inhibitor of TLR4) downregulated the serum levels of IL-6 and significantly alleviated inflammatory symptoms in the joint tissues on day 21 of treatment. Lu et al. (2021) demonstrated that celastrol treatment inhibited the release of inflammatory cytokines, such as TNF-α, IL-6, and IL-1β in the collagen-induced arthritis (CIA) animal model and suppressed RA-induced cellular autophagy by inhibiting the activation of the TLR2/HMGB1 signaling pathway.

In RA, TLR1 is expressed on fibroblasts (Ospelt and Gay 2010). Previous studies have demonstrated that TLR2 and TLR4 are upregulated in the macrophages and fibroblasts in the inner layer of ST of patients with RA (Huang et al. 2007; Iwahashi et al. 2004). In vitro studies have confirmed that the TLR2 and TLR4 expression levels were upregulated in peripheral blood monocytes, ST macrophages, and fibroblasts of patients with RA (Radstake et al. 2004), suggesting that TLR2 and TLR4 are associated with the pathogenesis of RA. IL-17 upregulates the TLR2, TLR3, and TLR4 expression levels in ST fibroblasts of patients with RA (Lee et al. 2014). The expression of TLR2 in ST fibroblasts of patients with RA is regulated by TNF, IL-1, LPS, and miR-19 (Iwahashi et al. 2004; Ospelt et al. 2008; Seibl et al. 2003; Jung et al. 2007; Meng et al. 2010; Sacre et al. 2007; Kim et al. 2007). Treatment with macrophage colony-stimulating factor and IL-10 upregulated the TLR2 expression levels in monocytes and ST macrophages of patients with RA (Iwahashi et al. 2004). In ST, articular fibroblasts exhibit upregulated levels of TLR3 during the early stages of RA (Ospelt et al. 2008). One study demonstrated that TLR3 expression is similar in the monocytes of patients with RA and healthy controls (Roelofs et al. 2009). IFNα upregulates TLR3 expression in myeloid cells and ST fibroblasts of patients with RA (Roelofs et al. 2009; Zhu et al. 2011), suggesting a close correlation between TLR3 and IFN-α in ST of patients with RA. TLR5 expression is reported to be upregulated in the inner layer fibroblasts and macrophages of patients with RA (Kim et al. 2014, 2013; Chamberlain et al. 2012), TNF and IL-17 upregulate the TLR5 expression levels in the peripheral blood mononuclear cells and differentiated macrophages of patients with RA in vitro. Meanwhile, multiple single factors upregulated TLR5 levels in ST fibroblasts of patients with RA. TLR5 expression was positively correlated with RA disease activity (DAS28) and TNF levels. Additionally, TLR5 expression in patients treated with antirheumatic drugs (DMARDs) and anti-TNF antibodies was significantly downregulated when compared with that in patients treated with DMARDs alone (Chamberlain et al. 2012). Pro-inflammatory cytokines, such as IL-1β, IL-17, IL-6, and IL-8 can significantly upregulate TLR5 expression in fibroblasts of patients with RA (Chamberlain et al. 2012). Previous studies have demonstrated that TLR7 and TLR8 are mainly expressed in ST-lining/sub-lining macrophages and ST-lining fibroblasts of patients with RA (Chamberlain et al. 2013). IL-17 and IL-8 can upregulate TLR7 expression. LPS and IL-1 upregulated the TLR8 levels in the monocytes and macrophages of patients with RA. TLR7 expression in monocytes of patients with RA is correlated with DAS28 and TNF levels, whereas TLR8 expression in the myeloid cells was not correlated with the DAS28 or TNF levels. TLR9 expression is upregulated in ST fibroblasts of patients with RA and can be induced by hypoxia (Hu et al. 2014). Additionally, the peripheral blood mononuclear cell and SF macrophage expression levels of TLR9 in patients with active RA were significantly higher than those in healthy control (Jongbloed et al. 2006). TLR9 (rs187084) single-nucleotide polymorphism (SNP) is correlated with RA susceptibility in the Turkish population (Etem et al. 2011). TNF suppression can significantly downregulate the expression levels of TLR1, TLR4, and TLR6 in the whole blood of CIA rats (Clanchy et al. 2021). TLR3 agonists can relieve arthritis by inhibiting synovial cell proliferation and inflammatory response (Yarilina et al. 2007). TLR4 antagonists alleviate joint inflammation in CIA rats by downregulating the IL-1 levels (Abdollahi-Roodsaz et al. 2007). The topical application of TLR5 agonists exacerbates joint inflammation and bone erosion (Kim et al. 2013; Chamberlain et al. 2012). Anti-TLR5 antibody treatment can reduce M1 macrophage and Th17 cell polarization (Kim et al. 2014). Hegewald et al. (2020), for the first time, demonstrated that enhanced osteoclast maturation is mediated by TLR7/8 signaling, which is activated by miR-574-5p. This is a novel mechanism for the involvement of small extracellular vesicles and miRNAs in RA pathogenesis, suggesting that miR-574-5p inhibitors can exert protective effects against osteoclast-mediated bone destruction in RA and inhibit bone resorption. The administration of TLR9 agonists relieves joint inflammation by promoting IL-12 and IFN-γ production and inhibiting synovial neutrophil infiltration (Wu et al. 2007). Data from a machine learning model revealed the correlation between TLR9 polymorphism (rs352139) and treatment response in patients with RA who were treated with TNF-α inhibitors (Kim et al. 2021).

### Systemic lupus erythematosus

Systemic lupus erythematosus (SLE), an autoimmune disease closely associated with HMGB1 and TLRs, is characterized by autoantibody production and systemic inflammation involving multiple organ systems (Tamirou et al. 2018). Previous studies have demonstrated that HMGB1 levels are upregulated in the blood of patients with SLE and are correlated with disease activity (Jiang and Pisetsky 2008; Ma et al. 2012; Li et al. 2010). The circulating immune complexes in patients with SLE also contain HMGB1, which is required to activate the immune response. In skin lesions of patients with SLE, the expression of HMGB1, which is expressed in the cytoplasmic and extracellular spaces, is upregulated in the epidermis and dermis and is correlated with the levels of IL-1β and TNF (Ardoin and Pisetsky 2008; Popovic et al. 2005). Ultraviolet radiation increases the translocation of HMGB1 into the cytoplasm and extracellular spaces in the skin of patients with SLE (Barkauskaite et al. 2007), suggesting that photosensitivity can induce SLE through HMGB1. The HMGB1-DNA complex can induce the production of anti-DNA antibodies in mice under the condition of DNA inactivation alone. The production of this antibody is dependent on TLR2 (Urbonaviciute et al. 2008).

TLRs are also involved in the pathogenesis of SLE. The peripheral blood mononuclear cell expression levels of TLR2 and TLR4 in patients with SLE are higher than those in the healthy control group (Komatsuda et al. 2008; Lee et al. 2016). In the lupus-prone model, TLR2 or TLR4 knockdown downregulated the antinuclear antibody levels and alleviated disease symptoms (Lartigue et al. 2009). TLR4 ligand endotoxin promotes the progression of lupus nephritis and autoantibody production (Cavallo and Granholm 1990a, b; Liu et al. 2006). TLR7 overexpression promoted autoantibody production (Pisitkun et al. 2006; Subramanian et al. 2006). In contrast, in the absence of TLR7, the levels of circulating autoantibodies and inflammatory cytokines, such as IL-6 and INF-α were significantly downregulated and the disease symptoms were alleviated (Christensen et al. 2006; Lee et al. 2008; Kono et al. 2009). TLR7 is closely correlated with the expression of IFN-α and IFN-β in patients with SLE (Paradowska-Gorycka et al. 2021). TLR8 overexpression is associated with the progression of SLE glomerulonephritis (Kimura et al. 2014). Estrogen treatment stimulates STAT1-dependent transcriptional activation of TLR8 (Young et al. 2014, 2017), suggesting that estrogen application may contribute to the progression of SLE. Some studies found that in the lupus-prone model, TLR9 knockdown did not improve the disease status and promoted disease progression (Christensen et al. 2006; Nickerson et al. 2010; Jackson et al. 2014), suggesting that TLR9 may exert a positive regulatory effect on the development of SLE. In the absence of TLR9, autoimmune disease is exacerbated, resulting in heightened activation of lymphocytes and plasmacytoid DC, as well as elevated levels of serum IgG and IFN-α (Christensen et al. 2006), suggesting the activation of TLR9 may be potential therapeutic strategies for SLE. A recent study has identified a mechanism through which TLR9 hampers the progression of SLE. The researchers observed that TLR9^P915H^ (TLR9 point mutant—lacking MyD88), in contrast to TLR9^K51E^, exhibited a more pronounced inhibition of disease progression compared to TLR9^WT^, and TLR9^P915H^ mice exhibited less disease than TLR9^K51E^ mice (TLR9 point mutant—lacking either ligand) (Leibler et al. 2022). These findings suggest that the anti-inflammatory response is encoded by a signal dependent on the TLR9 ligand, rather than MyD88. However, the study also reveals an unexpected pro-inflammatory effect of TLR9-MyD88 signaling, despite TLR9's primary role being thought to prevent SLE (Leibler et al. 2022). The study found that selective elimination of TLR9-MyD88 (TLR9^P915H^) signaling reduced disease compared to WT mice.

Ji et al. reported that HMGB1 expression was significantly upregulated in the bone marrow of patients with SLE. The clinical signs of lupus nephritis were alleviated and the survival time was prolonged in MRL/LPR mice administered with the safe HMGB1 inhibitor ethyl pyruvate (EP) for 8 weeks (Ji et al. 2019), suggesting that EP is a potential therapeutic for SLE. The benzylamine derivative FC-99 inhibited the TLR ligand-induced activation of pro-inflammatory cytokines, such as IL-12 and CXCL10, alleviated albuminuria, and upregulated immunoglobulin levels associated with renal disease and lupus-like syndrome in vitro and in vitro (Gao et al. 2017).

### Type 1 diabetes mellitus

Type 1 diabetes mellitus (T1DM), an autoimmune disease, is characterized by the progressive destruction of pancreatic β-cells mediated by immune cells. During autoimmune responses, HMGB1 can be passively released from damaged pancreatic cells and actively secreted by islet-infiltrating immune cells. The suppression of HMGB1 significantly inhibited the progression of diabetes in mice (Han et al. 2008). The serum HMGB1 levels were significantly upregulated in patients with T1DM and animal models (Han et al. 2008; Zhang et al. 2009; Wu et al. 2016). In T1DM, extracellular HMGB1 promotes autoimmune response by activating TLR4 and destabilizing and disrupting the function of regulatory T cells (Zhang et al. 2020). The upregulation of circulating HMGB1 in patients with T1DM leads to the instability of T regulatory cells, suggesting that blocking HMGB1 may be an effective therapeutic approach for T1DM (Zhang et al. 2020).

The peripheral blood mononuclear cell surface expression levels of TLR2 and TLR4 in patients with T1DM were significantly higher than those in the healthy control group. Additionally, the expression levels of TLRs, MyD88, TRIF, and downstream proteins were significantly upregulated in patients with T1DM. Furthermore, the secretory levels of IL-1 and TNF-α from the peripheral blood mononuclear cells were significantly upregulated in patients with T1DM and positively correlated with the TLR2 and TLR4 expression levels (Devaraj et al. 2008). Previous studies have reported that the inhibition of TLR2 or TLR4 in diabetes suppresses the inflammatory response (Lin et al. 2012; Ma et al. 2014; Jialal et al. 2014).

Continuous intake of glycyrrhizin acid after the onset of diabetes significantly downregulates retinal HMGB1 expression (Abu El-Asrar et al. 2014). Resveratrol, a dietary antioxidant, prevents morphological and functional ventricular remodeling and downregulates HMGB1 expression in T1DM rats (Delucchi et al. 2012). Sodium butyrate inhibited the expression of HMGB1 and NF-κB proteins in the pancreas and suppressed the progression of T1DM by inhibiting HMGB1 and downregulating the NF-κB-mediated inflammatory signaling pathways (Guo et al. 2018). Insulin infusion inhibits the expression of HMGB1 and TLRs in monocytes of patients with T1DM (Dandona et al. 2013). Further studies are needed to identify other drugs that can delay the progression of T1DM or its complications by inhibiting HMGB1 or TLRs.

## Autoimmune thyroid disease

HMGB1 may also be involved in the pathogenesis of Autoimmune Thyroid Disease (AITD). AITD, including Hashimoto's thyroiditis (HT) and Graves' disease (GD), are organ-specific autoimmune diseases characterized by lymphocytic infiltration of the thyroid gland (Lee et al. 2015). HMGB1 expression is upregulated in the peripheral blood of patients with AITD (including patients with HT or GD) (Peng et al. 2016), the thyroid tissue and serum of NOD.H-2^h4^ mice (murine model of autoimmune thyroiditis) (Li et al. 2017), the thyroid tissue and serum of a rat model of thyroglobulin-induced experimental autoimmune thyroiditis (EAT) (Guo et al. 2021), and thyroid follicular epithelial cells (Guo et al. 2021). The upregulated HMGB1 expression was positively correlated with thyroglobulin antibody and thyroid peroxidase antibody levels in the peripheral blood of patients with AITD (Peng et al. 2016). Previous studies have demonstrated that the number of HMGB1-positive monocytes is upregulated in patients with GD and that HMGB1 expression is downregulated by approximately 50% upon treatment with antithyroid drug (Mobarrez et al. 2016). These findings suggest that HMGB1 may play an important role in the development of AITD and that it is associated with disease progression.

The expression levels of TLR2, TLR3, TLR9, and TLR10 in peripheral blood monocytes of AITD patients were up-regulated (Peng et al. 2016). Additionally, the expression levels of TLR2, TLR3, TLR9, and the downstream adaptor protein MyD88 were significantly upregulated in the thyroid tissue and serum of NOD.H-2^h4^ mice or EAT rats (Li et al. 2017; Guo et al. 2021; Harii et al. 2005), as well as in thyroid follicular epithelial cells (Zhang et al. 2022). This suggests that TLR2, TLR3, and TLR9 are involved in the development of AITD. HMGB1 expression was upregulated in the peripheral blood mononuclear cells of patients with AITD both at resting state and upon TLR9 stimulation (Peng et al. 2016). This indicates a positive correlation between HMGB1 and TLR9 expression. Thus, HMGB1 plays an important role in the development of chronic inflammation in AITD by activating the TLR9 pathway. TLR10 SNP is significantly associated with the pathogenesis of AITD (Cho et al. 2015). Additionally, TLR10 is suggested to function as a co-receptor for TLR2 (Guan et al. 2010). However, the role of TLR10 in thyroid cells must be examined further.

HMGB1 promotes the expression of early pro-inflammatory cytokines, such as TNF-α, IL-1β, and IL-6 (Li et al. 2017). The inhibition of HMGB1 using glycyrrhizin (GL) suppresses thyroid lymphocyte infiltration and inhibits the downstream proteins MyD88 and NF-κB and the expression of pro-inflammatory cytokines through the HMGB1-TLR2 signaling pathway (Li et al. 2017). These findings suggest that the GL-mediated inhibition of HMGB1 mitigates tissue damage by alleviating inflammatory response and that GL may inhibit the development of AITD by suppressing the HMGB1-TLR2 signaling pathway. Guo reported that *Prunella vulgaris* L. (PV)-mediated HMGB1 inhibition can suppress inflammatory response and cytokine production by inhibiting the expression of MyD88 (downstream protein of the HMGB1-TLR9 signaling pathway), decreasing the proportion of Th1, Th2, and Th17 cells in splenocytes, and reversing the enhanced production of pro-inflammatory cytokines, such as TNF-α, IL-6, and IL-1β in vivo and in vitro (Guo et al. 2021)*.* This suggests that PV can delay the progression of AITD by inhibiting the HMGB1-TLR9 signaling pathway.

HMGB1, TLR2, TLR3, TLR9, and TLR10 may aggravate tissue damage by promoting the release of pro-inflammatory cytokines and play a major role in the development of AITD. Further studies are needed to elucidate the specific roles of TLR3 and TLR10. GL can target the HMGB1-TLR2 signaling pathway, while PV can inhibit the HMGB1-TLR9 signaling pathway and downstream proteins to delay the progression of AITD. Further studies are needed to identify targeted therapeutic drugs.

## Conclusions and perspectives

HMGB1 is an early-warning protein that induces immune responses alone or in combination with TLRs. Clinical and experimental studies have demonstrated that HMGB1 and TLR-dependent pro-inflammatory mechanisms play a key role in the pathogenesis of autoimmune diseases. Thus, HMGB1 and TLRs are important biomarkers. Clinical trials on several drugs targeting HMGB1 and TLRs are currently ongoing. Targeting HMGB1 and TLRs for therapeutic purposes has shown promise, but there are several limitations and challenges that need to be considered. These include potential side effects and difficulties in developing effective drugs. One limitation is the pleiotropic nature of HMGB1 and TLRs signaling. Both HMGB1 and TLRs have diverse roles and functions in the immune system, inflammation, and various diseases. Modulating their activities may disrupt the delicate balance and potentially lead to adverse effects, including immune dysregulation, increased susceptibility to infections, or abnormal immune activation. Another challenge is the complexity of the HMGB1 and TLRs signaling networks. These pathways involve intricate molecular interactions and crosstalk with other signaling pathways. Designing drugs that specifically target HMGB1 or individual TLRs without affecting other related molecules requires a deep understanding of the underlying mechanisms. Moreover, there is a need for comprehensive preclinical and clinical studies to validate the safety and efficacy of HMGB1 and TLRs targeted therapies. In conclusion, while targeting HMGB1 and TLRs holds great therapeutic potential, it is crucial to address the limitations and challenges associated with their modulation.

Recent studies have demonstrated that HMGB1 and TLRs are closely related to inflammasomes, pyroptosis, and ferroptosis. The specific downstream pathways of HMGB1 and TLRs that lead to autoimmune and inflammatory diseases have also been gradually discovered. However, future studies must focus on distinguishing the origin of the positive feedback loop for HMGB1 (is it as inconclusive as the chicken or egg problem?), elucidating the mechanism through which HMGB1 enters the cells and binds to intracellular TLRs, and evaluating the therapeutic potential of HMGB1 and TLR antagonists in autoimmune disease as targeting HMGB1 and TLRs has been demonstrated to exert potent therapeutic effects on inflammatory and autoimmune diseases.

## Data Availability

Not applicable.

## Abbreviations

DAMP: Damage-associated molecular pattern
TLRs: Toll-like receptors
HMG: High-mobility group
RA: Rheumatoid arthritis
SLE: Systemic lupus erythematosus
AITD: Autoimmune thyroid disease
PAMPs: Pathogen-associated molecular models
HMGB: High-mobility group box
NESs: Nuclear export signals
NLSs: Nuclear localization signals
LPS: Lipopolysaccharide
PRRs: Pattern recognition receptors
TRIF: TIR domain-containing adaptor protein inducing IFNβ
TNF-α: Tumor necrosis factor-α
NF-κB: Nuclear factor kappa-light-chain-enhancer of activated B cells
IL: Interleukin
MyD88: Molecule myeloid differentiation primary response differentiation gene 88
IRFs: IFN regulatory factors (IRFs)
ST: Synovial tissue (ST)
CIA: Collagen-induced arthritis
SNP: Single-nucleotide polymorphism
EP: Ethyl pyruvate
HT: Hashimoto's thyroiditis
GD: Graves' disease
EAT: Experimental autoimmune thyroiditis
GL: Glycyrrhizin
PV: *Prunella vulgaris* L.
